# Voxel‐Based quantitative MRI reveals spatial patterns of grey matter alteration in multiple sclerosis

**DOI:** 10.1002/hbm.25274

**Published:** 2020-11-06

**Authors:** Emilie Lommers, Camille Guillemin, Gilles Reuter, Eve Fouarge, Gaël Delrue, Fabienne Collette, Christian Degueldre, Evelyne Balteau, Pierre Maquet, Christophe Phillips

**Affiliations:** ^1^ GIGA – CRC in vivo imaging University of Liège Liège Belgium; ^2^ Clinical Neuroimmunology Unit, Neurology Department CHU Liège Liège Belgium; ^3^ Psychology and Neuroscience of Cognition Research Unit University of Liège Liège Belgium; ^4^ Neurosurgery Department CHU Liège Liège Belgium; ^5^ GIGA – in silico medicine University of Liège Liège Belgium

**Keywords:** atrophy, demyelination, gray matter, multiple sclerosis, quantitative MRI, voxel‐based analysis

## Abstract

Despite robust postmortem evidence and potential clinical importance of gray matter (GM) pathology in multiple sclerosis (MS), assessing GM damage by conventional magnetic resonance imaging (MRI) remains challenging. This prospective cross‐sectional study aimed at characterizing the topography of GM microstructural and volumetric alteration in MS using, in addition to brain atrophy measures, three quantitative MRI (qMRI) parameters—magnetization transfer (MT) saturation, longitudinal (R1), and effective transverse (R2*) relaxation rates, derived from data acquired during a single scanning session. Our study involved 35 MS patients (14 relapsing–remitting MS; 21 primary or secondary progressive MS) and 36 age‐matched healthy controls (HC). The qMRI maps were computed and segmented in different tissue classes. Voxel‐based quantification (VBQ) and voxel‐based morphometry (VBM) statistical analyses were carried out using multiple linear regression models. In MS patients compared with HC, three configurations of GM microstructural/volumetric alterations were identified. (a) Co‐localization of GM atrophy with significant reduction of MT, R1, and/or R2*, usually observed in primary cortices. (b) Microstructural modifications without significant GM loss: hippocampus and paralimbic cortices, showing reduced MT and/or R1 values without significant atrophy. (c) Atrophy without significant change in microstructure, identified in deep GM nuclei. In conclusion, this quantitative multiparametric voxel‐based approach reveals three different spatially‐segregated combinations of GM microstructural/volumetric alterations in MS that might be associated with different neuropathology.

## Graphical abstract

This cross‐sectional study investigates the spatial distribution of grey matter microstructural damage in multiple sclerosis, using voxel‐based quantification of three MRI parameters, sensitive to myelin and iron contents: magnetization transfer saturation (MT sat), effective transverse (R2*) and longitudinal (R1) relaxation rates. Potential gray matter atrophy was also investigated by a concurrent voxel‐based morphometry analysis. We identify three different spatially segregated combinations of GM alterations, common to this MS patient cohort and potentially associated with different neuropathological characteristics: primary cortices, deep grey matter nuclei and hippocampus. The evidence of substantial demyelination of hippocampi beyond atrophic areas constitutes a key contribution of this study and usefully complements previous characterization of hippocampal damage in MS.

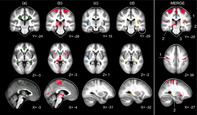

## 1 INTRODUCTION

Multiple sclerosis (MS) has long been considered as a disease of the sole white matter (WM). However, inflammation‐induced demyelination and neurodegeneration are also found in cortical and deep gray matters (GM), and expressed in different ways: focal lesions (i.e plaques) and diffuse alteration (Calabrese et al., 2015; Lassmann, 2018). Unfortunately, most GM damage escape detection on conventional MRI (Hulst & Geurts, 2011). Quantitative MRI (qMRI) potentially surmounts this limitation by quantifying physical properties of cerebral tissue and provides information about MS related microstructural alterations within GM. Magnetization transfer (MT) saturation, longitudinal (R1), and effective transverse (R2*) relaxation rates are differently sensitive to myelin and iron contents (Hametner et al., 2018; Schmierer, Scaravilli, Altmann, Barker, & Miller, 2004; Stüber et al., 2014) and were reported altered in MS cortical and deep GM (respectively CGM and DGM), taken as bulk tissue classes. In most studies, MT, R1, and R2* were found reduced in CGM of MS patients, suggesting reduction in myelin and/or iron contents (Filippi & Agosta, 2007; Lommers et al., 2019; Mainero et al., 2015; Vrenken et al., 2006). Their alteration in DGM structures seems more variable: MT is not consistently decreased (Filippi & Agosta, 2007; Lommers et al., 2019; Mainero et al., 2015; Vrenken et al., 2006) and R2* is either larger than (Elkady, Cobzas, Sun, Blevins, & Wilman, 2017; Khalil et al., 2011; Ropele et al., 2014) or not significantly different from healthy controls (HC) (Elkady et al., 2019; Hernández‐torres et al., 2018; Lommers et al., 2019). Furthermore, a few studies measuring R1 within DGM did not show any alteration of this parameter in MS population (Andica et al., 2019; Lommers et al., 2019).

Importantly most of these studies did not characterize the spatial distribution of GM microstructural alterations when considering several quantitative parameters. In this paper, we precisely provide a whole‐brain voxel‐based quantification (VBQ) of three qMRI parameters—MT saturation, R1 and R2*, derived from data acquired in a single MR session—and assess the spatial distribution of their changes in a cross‐sectional study which contrasted MS patients to HC. Potential GM atrophy was also investigated by a concurrent voxel‐based morphometry (VBM) analysis.

Our study aimed at characterizing the spatial distribution of microstructural and volumetric GM alterations induced by MS at the regional level (i.e., voxel‐wise). Results are presented as different spatial combinations of atrophy and microstructural damages in cortical and deep GM involvement, in MS compared with healthy controls.

## 2 MATERIALS AND METHODS

### 2.1 Participants

Seventy‐two participants were initially included in the study (Lommers et al., 2019): 36 patients with a diagnosis of MS according to McDonald criteria 2010 (15 relapsing–remitting MS, 14 primary progressive MS and 7 secondary progressive MS) and 36 healthy controls (HC), matched for age and gender, free from neurological or psychiatric disease. Patient inclusion criteria were: (a) age between 18 and 65 years, (b) Expanded Disability Status Scale (EDSS) ≤ 6.5; (c) absence of relapse within the previous 4 weeks; (d) compatibility with MRI. One relapsing–remitting MS patient was excluded because of an imperfect image preventing an optimal normalization of MRI data. Final demographic data are reported in Table 1. All participants were assessed clinically by a qualified MS specialist (EL) on the EDSS, Time 25‐Foot Walk, 9‐Hole Peg Test, oral Symbol Digit Modalities Test and California Verbal Learning Test. Scores for the last 4 tests were standardized to HC summary statistics and computed as a motor and cognitive composite scores (Lommers et al., 2019). This study was approved by the local ethic committee (B707201213806) and written informed consent was obtained from each participant.

**TABLE 1:** Participant characteristics

	HC (*n* = 36)	All patients (*n* = 35)	RRMS (*n* = 14, 40%)	PMS (*n* = 21, 60%)
**Age, y, mean (*SD*)**	45.86 (12.45)	46.2 (11.62)	37.14 (9.4)	52.23 (8.8)
**Sex (F/M)**	20/16	21/14	9/5	12/9
**Treatment** (first line/second line/non‐validated therapies)			9/5/0	2/3/2
**Disease duration, y, median (range)**	N/A	13 (0.5 to 35)	8 (0.5 to 28)	13 (2 to 35)
**Baseline EDSS, median (range)**		4 (1 to 6.0)	2 (1 to 5.5)	4.5 (3 to 6.0)
**Motor score, mean (*SD*)**	0.03 (0.72)	−2.32 (2.24)	−0.51 (1.21)	−3.39 (2)
**Cognitive score, mean (*SD*)**	0.04 (0.75)	−1.45 (1.74)	−0.22 (0.9)	−2.28 (1.68)
**Scanner 1/scanner 2**	11/25	25/10	10/4	15/6
**Volumetric data, %, mean (*SD*)**				
Gray matter fraction (GMF)	52.76 (1.99)	49.55 (3.02)^a^		
Lesion fraction	N/A	1.82 (1.4)		
**Median MPM values summarized over the whole tissue class, mean (*SD*)**				
***MT (p.u)***				
CGM	0.82 (0.09)	0.69 (0.27)^a^		
DGM	0.98 (1.13)	0.87 (0.11)^a^		
***R1 (Hz)***				
CGM	0.64 (0.02)	0.62 (0.02)^a^		
DGM	0.77 (0.06)	0.75 (0.05)^b^		
R2* *(Hz)*				
CGM	16.62 (1.02)	15.35 (1.16)^a^		
DGM	22.04 (3.10)	21.71 (2.9)^c^		

*Note:* ANOVA testing for differences between HC and MS patients.

Abbreviations: CGM, cortical gray matter; DGM, deep gray matter; GMF, gray matter fraction (GM/TIV volume); HC, healthy controls; LF, lesion fraction (lesion/TIV volume); PMS, progressive multiple sclerosis (primary and secondary PMS); RRMS, relapsing–remitting multiple sclerosis; TIV, total intracranial volume.

^a^ Differences statistically different at *p* < .0001.

^b^ Differences not statistically different, *p* = .06.

^c^ Differences not statistically different, *p* = .68.

### 2.2 MR image acquisition and spatial processing

MRI data were acquired on either of the following 3 T MRI‐scanners: Magnetom Allegra and Magnetom Prisma, Siemens Medical Solutions, Erlangen, Germany. The whole‐brain MRI acquisitions included a multiparameter mapping (MPM) protocol that has been gradually optimized and validated for multi‐centric acquisitions (Leutritz et al., 2020; Tabelow et al., 2019; Weiskopf et al., 2013). It consists of three co‐localized series of 3D multi‐echo fast low angle shot (FLASH) acquisitions at 1 × 1 × 1 mm^3^ resolution and two additional calibration sequences to correct for inhomogeneities in the RF transmit field (Lutti et al., 2012). The FLASH data sets were acquired with predominantly proton density (PD), T1, and MT weighting, referred to in the following as PDw, T1w and MTw echoes. Volumes were acquired in 176 sagittal slices using a 256 × 224 voxel matrix. Details of the MPM protocol used for this study are available as supplementary data. An additional FLAIR sequence was recorded with spatial resolution 1 × 1 × 1 mm^3^ and TR/TE/TI = 5,000 ms/516 ms/1800 ms.

**FIGURE 1:**
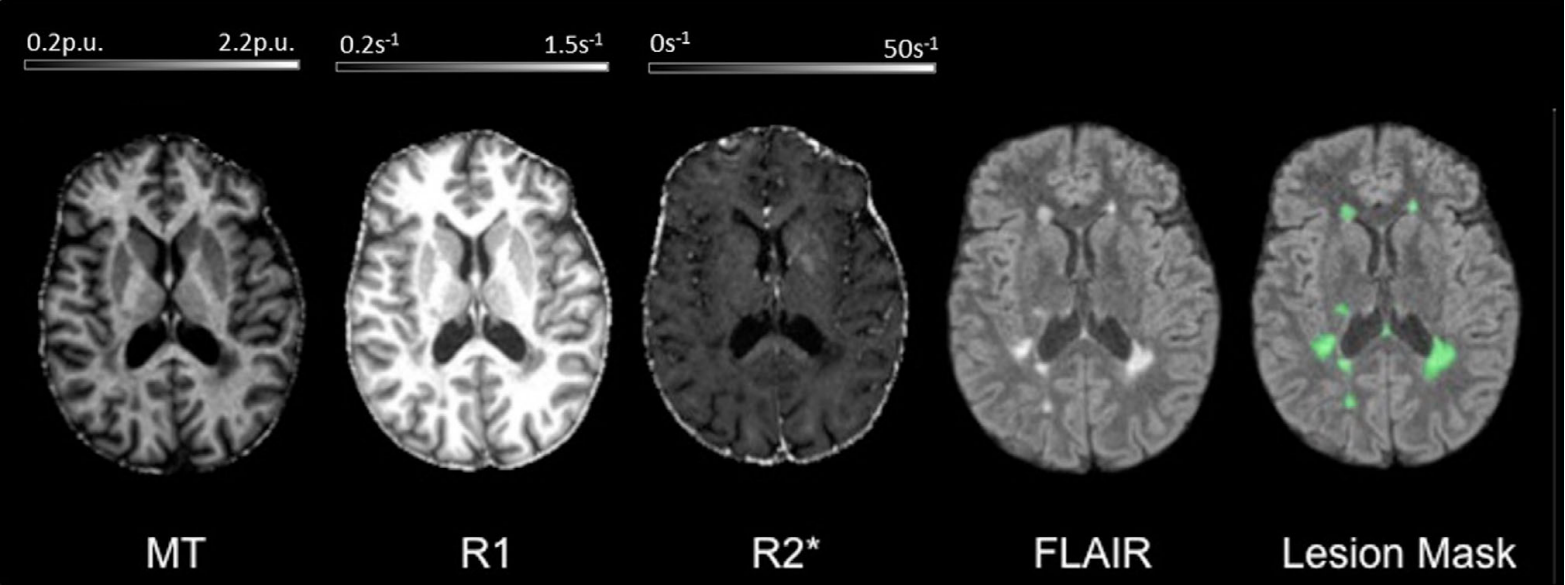
Example of MPM quantitative maps for a specific MS patient. From left to right: 3 MPM quantitative maps (MT, R1, R2*), standard FLAIR sequence image and FLAIR image overlaid with the estimated lesion mask. MT, magnetization transfer saturation; R1, longitudinal relaxation rate (1/T1); R2*, effective transverse relaxation rate (1/T2*); Lesion mask, posterior probability map of lesion tissue thresholded at 90%

All data analyses and processing were performed in Matlab (The MathWorks Inc., Natick, MA) using SPM12 (http://www.fil.ion.ucl.ac.uk/spm) and its extensions. MT saturation, R1 and R2* quantitative maps were estimated using the *hMRI* toolbox (http://hmri.info/) as previously described (Tabelow et al., 2019). Briefly, echoes for T1w, PDw, and MTw were extrapolated to TE = 0 to increase the signal‐to‐noise *ratio* and get rid of the otherwise remaining R2* bias (Tabelow et al., 2019). The resulting MTw and T1w (TE = 0) images were used to calculate MT saturation and R1 quantitative maps. To maximize the accuracy of the R1 and MT saturation maps, inhomogeneity in the flip angle was corrected by mapping the B1 transmit field according to the procedure detailed in (Lutti et al., 2012). In addition, intrinsically imperfect spoiling characteristics were accounted for and corrected in R1 map, using the approach described previously (Preibisch et al., 2009). The MT saturation map differs from the commonly used MT *ratio* (MTR, percent reduction in steady state signal) by explicitly accounting for spatially varying T1 relaxation time and flip angles. MT saturation shows a higher brain contrast to noise *ratio* than the MTR, leading to improved and more robust segmentation in healthy subjects (Helms, Dathe, & Dechent, 2010). The R2* map was estimated from all three multi‐echo series using the ESTATICS model (Tabelow et al., 2019). Example whole brain maps are shown in Figure 1. Note that these MR sequences at 3 T are not sensitive enough to detect focal cortical lesions, as previously described (Hulst & Geurts, 2011). Quantification of cortical parameters is thus possibly confounded by voxels located within cortical plaques.

MR images multi‐channel segmentation and normalization were performed with the standard “unified segmentation” (US) approach for the HC and its *US‐with‐Lesion* extension, accounting for WM lesions, for the MS patients. This part of the processing is largely detailed in a previous publication (Lommers et al., 2019). Briefly, for each MS patients, a preliminary lesion mask was derived from the FLAIR images and used to update the “tissue probability maps” with an extra lesion tissue class limited to the WM. This patient specific extended TPM was then used in the US tool, therefore accounting for the usual brain and head tissue plus the lesions (Phillips, Lommers, & Pernet, 2016). Individual lesion fraction (LF, *ratio* of WM lesion load to total intracranial volume) was computed afterwards from the segmented tissue classes. For VBM analyses, GM probability map (including cortical and deep GM) were spatially warped to standard space, modulated by the Jacobian determinants of the deformations, and smoothed with an isotropic Gaussian kernel (6 mm full width at half maximum—FWHM). For VBQ analyses, the 3 quantitative maps were normalized using the subject‐specific deformation field but without modulation. A tissue weighted smoothing (3 mm FWHM isotropic) yielded smoothed tissue‐specific multiparameter maps which optimally preserved quantitative parameter values within each tissue class (Draganski et al., 2011). Detailed analysis of the influence of spatial deformations onto quantitative parametric values proved this method to be largely insensitive to volumetric changes (i.e., atrophy) (Salvoni et al., 2019). Finally, a GM mask was generated: the smooth modulated warped individual GM, WM and CSF maps were averaged across all subjects and the GM mask included voxels for which mean GM probability was larger than that of WM or CSF and exceeded 20% (Callaghan et al., 2014).

### 2.3 Statistical analyses

Whole‐GM voxel‐wise VBM and VBQ statistical analyses, explicitly using the GM mask, were carried out using a multiple linear regression model embedded in the general linear model framework of SPM12. MRI data were analyzed in a factorial design, with the 2 different scanners as one factor and the group (MS vs. HC) as the second factor (Stonnington et al., 2008). Age, gender and total intracranial volume were entered as covariates of no interest. Differences between MS patients and HC as well as interactions between groups and scanners were tested by separate F‐tests for each quantitative parameter (MT, R1, R2*) and volume.

Post hoc *t* tests explored significant effects. Cluster‐level inferences were conducted at *p* < .05 after family‐wise error rate (FWER) correction for multiple comparisons across the whole GM (*p* < .0001 uncorrected cluster‐defining threshold). These 2‐sample t‐tests identified significant group effects, over and above the normal spatially heterogeneous distribution of quantitative parameters (Deistung et al., 2013) and accounting for potential unequal variance across groups.

In the patient population, three F tests looked for significant voxel‐wise regression between each qMRI parameter and clinical scores (EDSS, motor and cognitive composite scores) as well as lesion fraction. Significance threshold was set at *p* < .05 FWER corrected at cluster level (*p* < .0001 uncorrected cluster‐defining threshold).

## 3 RESULTS

**FIGURE 2:**
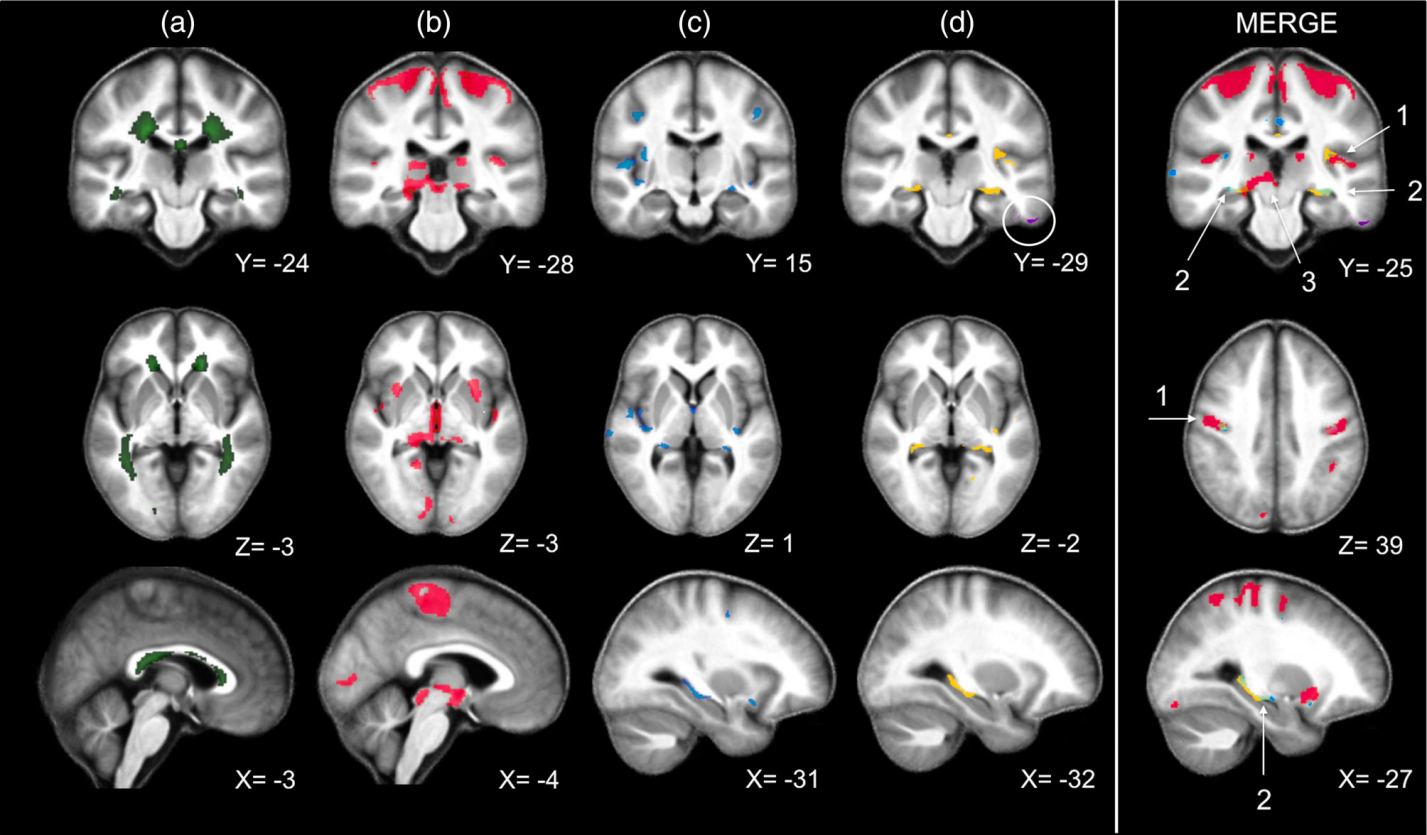
VBM and VBQ results superimposed on the group mean MT map. Green: Average WM lesion probability map of MS patients, thresholded at 90% (a); Voxels showing a significant difference between MS and HC (thresholded at cluster level, *p* < .05 FWER‐corrected): decreased gray matter (GM) volume in red (b), MT reduction in blue (c), R1 reduction in yellow and R2* reduction (circled) in violet (d). The right most column (MERGE) overlays the maps displayed in columns b, c, and d columns, same color scheme, and highlights the three different patterns discussed in the main text (1 = Primary Neocortical Regions, 2 = Hippocampus, 3 = Deep Gray Matter Nuclei). Images are shown in neurologic convention and the X/Y/Z coordinates indicate the slice position in millimeter in MNI space

Compared with HC, we identified significant loco‐regional reductions of MT saturation in GM of MS patients, bilaterally in Heschl's gyri, posterior hippocampi and precentral gyri. MT was also significantly reduced in right insula, right superior temporal gyrus, right angular gyrus, left caudate, and cingulum as well as left postcentral gyrus (Figure 2). R1 was significantly lower in MS patients, compared with HC, within both hippocampi, left temporal gyri (middle, superior, inferior), left insula, right sensory‐motor cortex, right middle temporal gyrus and right cingulum. Finally, we found significant clusters of locally reduced R2* in left inferior and middle temporal gyri as well as in left postcentral gyrus (Figure 2). We did not observe an increase of any of these three qMRI parameters in MS patients compared with HC. No significant parameter changes were observed between RRMS and PMS, probably due to the small number of observations.

Extensive GM loss was observed in MS compared with HC in bilateral precentral and Heschl's gyri, both thalami, both cunei, both putamen, supero‐inferior colliculi as well as in bilateral lingual gyri and right posterior hippocampus (Figure 2).

Regressions between GM qMRI values and clinical scores (EDSS, motor and cognitive composite scores) only showed that cognitive score decreased with regional reduction of R1 within right middle frontal gyrus. Furthermore MT, R1, and R2* negatively regressed with lesion fraction in both thalami and caudate nuclei.

**FIGURE 3:**
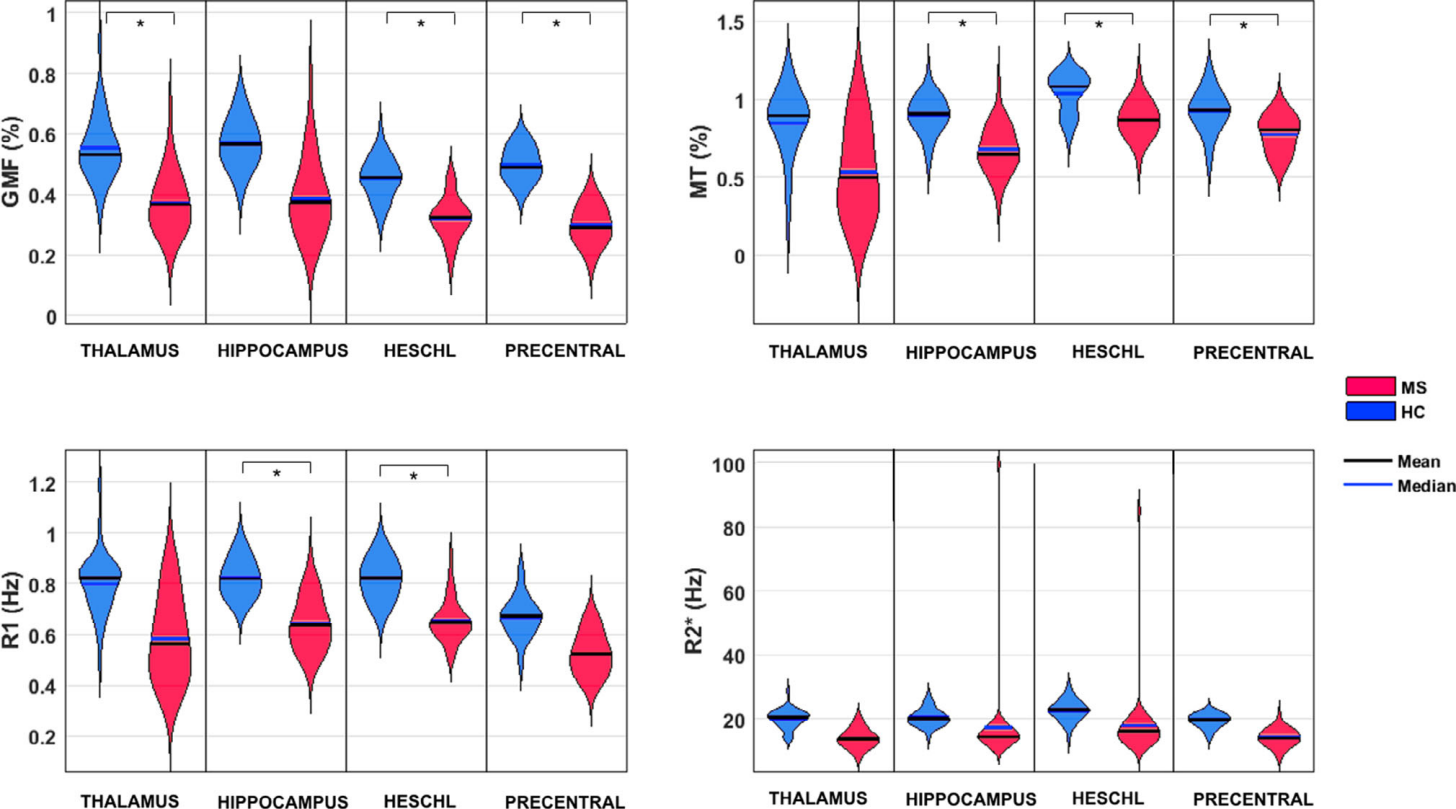
Illustration of the distribution of each GM parameter (GM volume, MT, R1, R2*) extracted from four different voxels, across MS and HC subjects in four brain regions of interest: Left thalamus (x = −15, y = −27, z = 14); Left hippocampus (x = −31, y = −27, z = −7); Left Heschl's gyrus (x = −37, y = −28, z = 10); Left precentral gyrus (x = −34, y = −6, z = 46). All voxels coordinates expressed in MNI space and chosen as the local statistical maximum in the ROI. Statistical significance (*) set at *p* < .05 FWER‐corrected for the whole GM volume

For a complete list of regions showing lower GM volume, lower MT, R1, R2* values and significant regressions, see Tables S1 to S6 in the supporting information section. Figure 3 illustrates the distribution of each GM parameter (GM volume, MT, R1, R2*) extracted from four different regions of interest (ROIs), across MS and HC participants.

Importantly, despite a clear effect of scanner on qMRI values (essentially MT saturation), there was no significant group by scan interaction, even at the set‐level.

## 4 DISCUSSION

The topography of microstructural and volumetric alterations in GM of MS patients was assessed by a multiparametric voxel‐based approach, without prior prediction regarding MS damage location. We reasoned that the simultaneous voxel‐wise quantification of physical tissue parameters would enrich the understanding of MS pathology by characterizing a typical microstructural and volumetric pattern, over and above the inter‐patient variability of disease presentation. Importantly, our quantification of microstructural parameters is largely insensitive to volumetric changes (Draganski et al., 2011; Salvoni et al., 2019), thereby allowing for independently characterizing microstructural and volumetric changes. Quantitative MRI parameters (MT, R1, R2*) inform us about GM microstructure (mainly myelin and iron contents) (Hametner et al., 2018; Schmierer et al., 2004; Stüber et al., 2014) while VBM estimates GM loss or atrophy secondary to neurono‐axonal loss and neuronal shrinkage (Klaver et al., 2015). Finally, lesion load in the underlying WM was taken into account to interpret GM alterations in parameter value (MT, R1, and R2*).

This multiparametric voxel‐based strategy has seldom been followed in MS: a single report on a cohort of 19 patients is available (Engström, Warntjes, Tisell, Landtblom, & Lundberg, 2014). Consequently, our results cannot be directly compared with the existing literature. A few voxel‐based studies examined the spatial distribution of MT *ratio* together with GM atrophy (Audoin et al., 2007; Crespy et al., 2011; Khaleeli et al., 2007; Mallik et al., 2015). Fairly consistent with those reported in other MS cohorts including RRMS and PMS patients (Bodini et al., 2009; Ceccarelli et al., 2008; Khaleeli et al., 2007; Mallik et al., 2015) our VBM results show significant GM loss essentially in primary motor cortices, Heschl's gyri, cunei and DGM nuclei (both thalami, both putamen, supero‐inferior colliculi). However, we did not find any significant atrophy within the cerebellum possibly because of an unsatisfactory GM segmentation within the posterior fossa. As for regions of lower MT saturation, they agree with previous reports regarding CGM but not DGM (Audoin et al., 2007; Khaleeli et al., 2007; Mallik et al., 2015). Again, comparison with literature is difficult because MT saturation estimated in the current work represents an advanced estimation of magnetization transfer over MT *ratio*. Moreover, one should keep in mind that we used fairly conservative statistical thresholds (to control for the risk of false positives) and that an absence of significant loco‐regional differences between HC and MS patients does not prove their absence: it might result from sparsely distributed, spatially variable across patients, areas of low focal MT, R1, or R2*.

Statistical inference identified three combinations of microstructural/volumetric changes (Figure 2): (a) Co‐localization of GM atrophy with microstructural changes, usually observed in areas overhanging the most abundant WM lesion load. Compared with HC, MS patients show a significant reduction in MT saturation and/or R1 (both sensitive to myelin content) co‐localized with widespread GM atrophy in bilateral sensory‐motor cortices, Heschl's gyri and right hippocampus. Significant R2* reduction is restricted to the left inferior and middle temporal gyri as well as to the left postcentral gyrus. (b) Microstructural modifications without significant GM loss: the left hippocampus and paralimbic cortices (cingulate gyrus and insula) show reduced MT and/or R1 values, without significant atrophy, suggesting a demyelination of residual neurono‐axonal tissue, a regional resilience to atrophy or the antecedence of microstructural alterations over neurodegeneration. (c) Significant atrophy without significant change in microstructure: this pattern was especially identified in DGM structures (thalami, putamen, supero‐inferior colliculi), suggesting the predominance of neurono‐axonal loss in these regions. The discrepancy between volumetric and microstructural changes highlights the complementarity of these MRI techniques in assessing GM pathological changes in MS.

### 4.1 Pattern 1: Primary neocortical regions

Several phenomena likely contribute to the combined demyelination and neurodegeneration observed in primary auditory and sensory‐motor cortices. These regions show less anatomical variability than other brain areas (e.g., prefrontal cortex), which facilitates the detection of commonalities across patients. They are connected with long‐range, densely myelinated tracts (Nieuwenhuys & Broere, 2017) that are vulnerable to trans‐synaptic—anterograde and retrograde—neurono‐axonal degeneration (Calabrese et al., 2015; Haider et al., 2016) supposedly because of their intrinsically heavy metabolic load (Calabrese et al., 2015) and their frequent involvement in focal WM inflammation. Finally, because of their numerous folds, these regions are more exposed to cerebrospinal fluid (CSF) stasis, supporting the hypothesis that soluble factors produced in the CSF by lymphocytes influence subpial demyelination, particularly in patients with progressive MS (Magliozzi et al., 2018).

### 4.2 Pattern 2: Hippocampus

The evidence of substantial demyelination of hippocampi beyond atrophic areas constitutes a key contribution of this study and usefully complements previous characterization of hippocampal damage in MS (Rocca et al., 2018). Indeed, demyelination is detected postmortem in 53 to 79% of MS hippocampi (Dutta et al., 2011; Dutta et al., 2013; Geurts et al., 2007) and recently in living patients by estimating GM myelin volume fraction with MRI (Andica et al., 2019). Hippocampal demyelination in MS is selectively associated with alterations in genic expression profiles, triggering abnormalities in hippocampal axonal traffic, synaptic plasticity, neurotransmitter homeostasis and memory (Dutta et al., 2011; Dutta et al., 2013). This stresses the need for early and specific MRI biomarkers for demyelination in MS. The mechanisms underpinning the relative resilience of hippocampus to atrophy in MS are beyond the scope of this study. Nevertheless, a few observations are consistent with our results. First, neuronal loss is inconsistently observed in demyelinated hippocampi while synaptic density is systematically decreased (Dutta et al., 2011; Geurts et al., 2007; Papadopoulos et al., 2009). By the same token, chronic inflammation potentially enhances neurogenesis within dentate gyrus (Rocca et al., 2015). Although the functional significance of these cellular changes is still under debate (Pluchino et al., 2008; Zhao, Deng, & Gage, 2008), they may balance neuronal loss, at the structural level (Rocca et al., 2015).

### 4.3 Pattern 3: Deep gray matter nuclei

Our results show a significant atrophy of DGM in MS and agree with previous reports (Hulst & Geurts, 2011). Thalamic and putamen atrophy relates to significant neuronal and axonal loss. It occurs very early in the disease course and exceeds cortical atrophy (Eshaghi et al., 2018). Due its extensive reciprocal connections with cortical and subcortical structures, thalami are particularly vulnerable to anterograde and retrograde degeneration. This interpretation is supported by the significant inverse relationship between each qMRI parameter value (MT, R1, and R2*) within thalami and the lesion load, which suggests that lesions in connecting WM tracts also alter thalamic microstructure. These neurodegenerative processes likely dominate local inflammatory activity and oxidative injury which were also reported (Haider et al., 2016) but were not sensitively assessed in this study.

### 4.4 Limitations

This cross‐sectional study was run on a relatively small sample size. To preserve statistical power, the two MS phenotypes were pooled together. We cannot rule out that results are partly driven by the larger proportion of PMS over RRMS patients, although exploratory tests did not show any significant difference between the two patient groups when corrected for multiple comparisons. If considering results significant at *p* < .001 uncorrected for multiple comparisons, PMS patients showed reduction in MT, R1, and R2* in a number of regions that were already detected in the contrast involving healthy control and the whole MS population. This might indicate that demyelination is even more severe as disease progresses. Because differences in microstructure between RRMS and PMS patients are of paramount importance, they will be assessed in future work, based on larger and independent population samples.

The factorial design used in this study offers the possibility to test separately the effect of disease and the effect of scanner, as well as their interaction. The absence of significant group by scan interaction allows us to disentangle the potential confound introduced by the two different scanners and still reliably discuss the effect of disease on GM microstructure. Furthermore, acquisition protocol has been optimized, pointing out the opportunity for multi‐centric studies (Leutritz et al., 2020).

Finally, our results do not confirm previous reports linking thalamic and hippocampal damage to motor performance and cognitive dysfunction in MS patients (Eshaghi et al., 2018; Rocca et al., 2018). Inferences were conservatively made after correction for multiple comparisons over the whole GM, increasing the risk of Type II error. In this preliminary study, we indeed considered that conservative inferences had to be preferred to spurious results. Alternatively, it might be the case that microstructural alterations precede the occurrence of clinical symptom: longitudinal studies are needed to answer this question. Moreover, spinal cord lesions were not taken into account although they impact motor performance.

## 5 CONCLUSION

This multiparametric voxel‐based approach identifies three different spatially‐segregated patterns of GM microstructural/volumetric alterations in MS patients, that might be associated with different neuropathology. The results highlight the usefulness of qMRI parameters and their complementarity with volumetric techniques in assessing GM status in MS.

## CONFLICT INTERESTS

The author(s) declared no potential conflicts of interest with respect to the research, authorship, and/or publication of this article.

## AUTHOR CONTRIBUTIONS

Emilie Lommers, Evelyne Balteau, Christophe Phillips, and Pierre Maquet contributed to the study concept and design. Emilie Lommers, Gilles Reuter, Camille Guillemin, Fabienne Collette, Christian Degueldre, Evelyne Balteau, Pierre Maquet, and Christophe Phillips contributed to MRI data acquisition and analysis. Emilie Lommers, Christophe Phillips and Pierre Maquet drafted the manuscript and figures.

## Supporting information


**Table S1** Regions with significantly reduced gray matter MT saturation in MS patients compared to healthy controls.
Tables S2: Regions with significantly reduced gray matter R1 in MS patients compared to healthy controls.
Tables S3: Regions with significantly reduced gray matter R2* in MS patients compared to healthy controls.
Tables S4: Regions with significantly reduced gray matter volume (VBM) in MS patients compared to healthy controls.
Tables S5: Clusters where regional MT, R1 and R2* decrease with lesion fraction.
Table S6: Clusters where cognitive score decreases with regional reduction of R1.

## Data Availability

The data that support the findings of this study are available on request from the corresponding author. The data are not publicly available due to privacy or ethical restrictions.

## Appendix A. Multi‐echo 3D FLASH acquisition parameters

**Table:**

	Magnetom ALLEGRA	Magnetom PRISMA
PDw		
TR	23.7 (ms)	24.5 (ms)
Flip angle	6°	6°
Bipolar gradient echoes/TE	6/TE 2.2–14.7 (ms)	8/TE 2.34–18.72 (ms)
T1w		
TR	18.7 (ms)	24.5 (ms)
Flip angle	20°	21°
Bipolar gradient echoes/TE	6/TE 2.2–14.7 (ms)	8/TE 2.34–18.72 (ms)
MTw		
TR	23.7 (ms)	24.5 (ms)
Flip angle	6°	6°
Bipolar gradient echoes/TE	6/TE 2.2–14.7 (ms)	6/TE 2.34–14.04 (ms)
Bandwidth	425 (Hz/Px)	465 (Hz/Px)
Off‐resonance Gaussian MT pulse	FA: 215° Frequency offset: 2(kHz)	FA: 220° Frequency offset: 2(kHz)

## References

[hbm25274-bib-0001] Andica, C. , Hagiwara, A. , Kamagata, K. , Yokoyama, K. , Shimoji, K. , Saito, A. , … Aoki, S. (2019). Gray matter alterations in early and late relapsing‐remitting multiple sclerosis evaluated with synthetic quantitative magnetic resonance imaging. Scientific Reports, 9, 1–10. 10.1038/s41598-019-44615-3 31148572 PMC6544650

[hbm25274-bib-0002] Audoin, B. , Davies, G. , Rashid, W. , Fisniku, L. , Thompson, A. J. , & Miller, D. H. (2007). Voxel‐based analysis of grey matter magnetization transfer ratio maps in early relapsing remitting multiple sclerosis. Multiple Sclerosis, 13, 483–489. 10.1177/1352458506070450 17463071

[hbm25274-bib-0003] Bodini, B. , Khaleeli, Z. , Cercignani, M. , Miller, D. H. , Thompson, A. J. , & Ciccarelli, O. (2009). Exploring the relationship between white matter and gray matter damage in early primary progressive multiple sclerosis: An in vivo study with TBSS and VBM. Human Brain Mapping, 30, 2852–2861. 10.1002/hbm.20713 19172648 PMC6871131

[hbm25274-bib-0004] Calabrese, M. , Magliozzi, R. , Ciccarelli, O. , Geurts, J. J. G. , Reynolds, R. , & Martin, R. (2015). Exploring the origins of grey matter damage in multiple sclerosis. Nature Reviews. Neuroscience, 16, 147–158. 10.1038/nrn3900 25697158

[hbm25274-bib-0005] Callaghan, M. F. , Freund, P. , Draganski, B. , Anderson, E. , Cappelletti, M. , Chowdhury, R. , … Weiskopf, N. (2014). Widespread age‐related differences in the human brain microstructure revealed by quantitative magnetic resonance imaging. Neurobiology of Aging, 35, 1862–1872. 10.1016/j.neurobiolaging.2014.02.008 24656835 PMC4024196

[hbm25274-bib-0006] Ceccarelli, A. , Rocca, M. A. , Pagani, E. , Colombo, B. , Martinelli, V. , Comi, G. , & Filippi, M. (2008). A voxel‐based morphometry study of grey matter loss in MS patients with different clinical phenotypes. NeuroImage, 42, 315–322. 10.1016/j.neuroimage.2008.04.173 18501636

[hbm25274-bib-0007] Crespy, L. , Zaaraoui, W. , Lemaire, M. , Rico, A. , Faivre, A. , Malikova, I. , … Audoin, B. (2011). Prevalence of grey matter pathology in early multiple sclerosis assessed by magnetization transfer ratio imaging. PLoS One, 6, 2–7. 10.1371/journal.pone.0024969 PMC3174243 21949813

[hbm25274-bib-0008] Deistung, A. , Schäfer, A. , Schweser, F. , Biedermann, U. , Turner, R. , & Reichenbach, J. R. (2013). Toward in vivo histology: A comparison of quantitative susceptibility mapping (QSM) with magnitude‐, phase‐, and R2*‐imaging at ultra‐high magnetic field strength. NeuroImage, 65, 299–314. 10.1016/j.neuroimage.2012.09.055 23036448

[hbm25274-bib-0009] Draganski, B. , Ashburner, J. , Hutton, C. , Kherif, F. , Frackowiak, R. S. J. J. , Helms, G. , & Weiskopf, N. (2011). Regional specificity of MRI contrast parameter changes in normal ageing revealed by voxel‐based quantification (VBQ). NeuroImage, 55, 1423–1434. 10.1016/j.neuroimage.2011.01.052 21277375 PMC3093621

[hbm25274-bib-0010] Dutta, R. , Chang, A. , Doud, M. K. , Kidd, G. J. , Ribaudo, M. V. , Young, E. A. , … Trapp, B. D. (2011). Demyelination causes synaptic alterations in hippocampi from multiple sclerosis patients. Annals of Neurology, 69, 445–454. 10.1002/ana.22337 21446020 PMC3073544

[hbm25274-bib-0011] Dutta, R. , Chomyk, A. M. , Chang, A. , Ribaudo, M. V. , Deckard, S. A. , Doud, M. K. , … Trapp, B. D. (2013). Hippocampal demyelination and memory dysfunction are associated with increased levels of the neuronal microRNA miR‐124 and reduced AMPA receptors. Annals of Neurology, 73, 637–645. 10.1002/ana.23860 23595422 PMC3679350

[hbm25274-bib-0012] Elkady, A. M. , Cobzas, D. , Sun, H. , Blevins, G. , & Wilman, A. H. (2017). Progressive iron accumulation across multiple sclerosis phenotypes revealed by sparse classification of deep gray matter. Journal of Magnetic Resonance Imaging, 46, 1464–1473. 10.1002/jmri.25682 28301067

[hbm25274-bib-0013] Elkady, A. M. , Cobzas, D. , Sun, H. , Seres, P. , Blevins, G. , & Wilman, A. H. (2019). Five year iron changes in relapsing‐remitting multiple sclerosis deep gray matter compared to healthy controls. Multiple Sclerosis and Related Disorders, 33, 107–115. 10.1016/j.msard.2019.05.028 31181540

[hbm25274-bib-0014] Engström, M. , Warntjes, J. B. M. , Tisell, A. , Landtblom, A.‐M. , & Lundberg, P. (2014). Multi‐parametric representation of voxel‐based quantitative magnetic resonance imaging. PLoS One, 9, e111688 10.1371/journal.pone.0111688 25393722 PMC4230947

[hbm25274-bib-0015] Eshaghi, A. , Brownlee, W. J. , Altmann, D. R. , Tur, C. , Cardoso, M. J. , De Angelis, F. , … Alexander, D. C. (2018). Deep gray matter volume loss drives disability worsening in multiple sclerosis. Annals of Neurology, 83, 210–222. 10.1002/ana.25145 29331092 PMC5838522

[hbm25274-bib-0016] Filippi, M. , & Agosta, F. (2007). Magnetization transfer MRI in multiple sclerosis. Journal of Neuroimaging, 17(Suppl 1), 22S–26S. 10.1111/j.1552-6569.2007.00132.x 17425730

[hbm25274-bib-0017] Geurts, J. J. G. , Bo, L. , Roosendaal, S. D. , Hazes, T. , Barkhof, F. , Witter, M. P. , … Van Der Valk, P. (2007). Extensive hippocampal demyelination in multiple sclerosis. Journal of Neuropathology and Experimental Neurology, 66, 819–827. 10.1097/nen.0b013e3181461f54 17805012

[hbm25274-bib-0018] Haider, L. , Zrzavy, T. , Hametner, S. , Ho, R. , Trattnig, S. , Pfeifenbring, S. , … Bru, W. (2016). The topograpy of demyelination and neurodegeneration in the multiple sclerosis brain. Brain, 139, 807–815. 10.1093/brain/awv398 26912645 PMC4766379

[hbm25274-bib-0019] Hametner, S. , Endmayr, V. , Deistung, A. , Palmrich, P. , Prihoda, M. , Haimburger, E. , … Grabner, G. (2018). The influence of brain iron and myelin on magnetic susceptibility and effective transverse relaxation ‐ a biochemical and histological validation study. NeuroImage, 179, 117–133. 10.1016/j.neuroimage.2018.06.007 29890327

[hbm25274-bib-0020] Helms, G. , Dathe, H. , & Dechent, P. (2010). Modeling the influence of TR and excitation flip angle on the magnetization transfer ratio (MTR) in human brain obtained from3D spoiled gradient echoMRI. Magnetic Resonance in Medicine, 64, 77–185. 10.1002/mrm.22379 20572139

[hbm25274-bib-0021] Hernández‐torres, E. , Wiggermann, V. , Machan, L. , Sadovnick, A. D. , Li, D. K. B. , Traboulsee, A. , … Rauscher, A. (2018). Increased mean R2 * in the deep gray matter of multiple sclerosis patients: Have we been measuring atrophy? Journal of Magnetic Resonance Imaging, 50, 1–8. 10.1002/jmri.26561 30511803

[hbm25274-bib-0022] Hulst, H. E. , & Geurts, J. J. G. (2011). Gray matter imaging in multiple sclerosis: What have we learned ? BMC Neurology, 11(153), 1–11. 10.1186/1471-2377-11-153 22152037 PMC3262750

[hbm25274-bib-0023] Khaleeli, Z. , Cercignani, M. , Audoin, B. , Ciccarelli, O. , Miller, D. H. , & Thompson, A. J. (2007). Localized grey matter damage in early primary progressive multiple sclerosis contributes to disability. NeuroImage, 37, 253–261. 10.1016/j.neuroimage.2007.04.056 17566765

[hbm25274-bib-0024] Khalil, M. , Langkammer, C. , Ropele, S. , Petrovic, K. , Wallner‐Blazek, M. , Loitfelder, M. , … Fazekas, F. (2011). Determinants of brain iron in multiple sclerosis: A quantitative 3T MRI study. Neurology, 77, 1691–1697. 10.1212/WNL.0b013e318236ef0e 21975210

[hbm25274-bib-0025] Klaver, R. , Popescu, V. , Voorn, P. , Galis‐de Graaf, Y. , van der Valk, P. , de Vries, H. E. , … Geurts, J. J. G. (2015). Neuronal and axonal loss in normal‐appearing gray matter and subpial lesions in multiple sclerosis. Journal of Neuropathology and Experimental Neurology, 74, 453–458. 10.1097/NEN.0000000000000189 25853695

[hbm25274-bib-0026] Lassmann, H. (2018). Multiple sclerosis pathology. Cold Spring Harb Perspect, 8, a028936 10.1101/cshperspect.a028936 PMC5830904 29358320

[hbm25274-bib-0027] Leutritz, T. , Samson, R. S. , Curt, A. , Helms, G. , Freund, P. , & Weiskopf, N. (2020). Multiparameter mapping of relaxation ( R1, R2 *), proton density and magnetization transfer saturation at 3 T: A multicenter dual‐vendor reproducibility and repeatability study. Human Brain Mapping, 41, 1–16. 10.1002/hbm.25122 PMC7502832 32639104

[hbm25274-bib-0028] Lommers, E. , Simon, J. , Reuter, G. , Delrue, G. , Dive, D. , Degueldre, C. , … Maquet, P. (2019). Multiparameter MRI quantification of microstructural tissue alterations in multiple sclerosis. NeuroImage Clinic, 23, 101879 10.1016/j.nicl.2019.101879 PMC6555891 31176293

[hbm25274-bib-0029] Lutti, A. , Stadler, J. , Josephs, O. , Windischberger, C. , Speck, O. , Bernarding, J. , … Weiskopf, N. (2012). Robust and fast whole brain mapping of the RF transmit field B1 at 7T. PLoS One, 7, 1–7. 10.1371/journal.pone.0032379 PMC3299646 22427831

[hbm25274-bib-0030] Magliozzi, R. , Howell, O. W. , Nicholas, R. , Cruciani, C. , Castellaro, M. , Romualdi, C. , … Pizzini, F. B. (2018). Inflammatory intrathecal profiles and cortical damage in multiple sclerosis. Annals of Neurology, 83, 739–755. 10.1002/ana.25197 29518260

[hbm25274-bib-0031] Mainero, C. , Louapre, C. , Govindarajan, S. T. , Gianni, C. , Scott Nielsen, A. , Cohen‐adad, J. , … Kinkel, R. P. (2015). A gradient in cortical pathology in multiple sclerosis by in vivo quantitative 7 T imaging. Brain, 138, 932–945. 10.1093/brain/awv011 25681411 PMC4677339

[hbm25274-bib-0032] Mallik, S. , Muhlert, N. , Samson, R. S. , Sethi, V. , Wheeler‐kingshott, C. A. M. , Miller, D. H. , & Chard, D. T. (2015). Regional patterns of grey matter atrophy and magnetisation transfer ratio abnormalities in multiple sclerosis clinical subgroups: A voxel‐based analysis study. Multiple Sclerosis Journal, 21, 423–432. 10.1177/1352458514546513 25145689 PMC4390521

[hbm25274-bib-0033] Nieuwenhuys, R. , & Broere, C. A. J. (2017). A map of the human neocortex showing the estimated overall myelin content of the individual architectonic areas based on the studies of Adolf Hopf. Brain Structure & Function, 222, 465–480. 10.1007/s00429-016-1228-7 27138385 PMC5225164

[hbm25274-bib-0034] Papadopoulos, D. , Dukes, S. , Patel, R. , Nicholas, R. , Vora, A. , & Reynolds, R. (2009). Substantial archaeocortical atrophy and neuronal loss in multiple sclerosis. Tissue Samples. Brain Pathology, 19, 238–253. 10.1111/j.1750-3639.2008.00177.x PMC8094861 18492094

[hbm25274-bib-0035] Phillips, C. , Lommers, E. , & Pernet, C. (2016). Unifying lesion masking and tissue probability maps for improved segmentation and normalization Paper presented at 23rd annual meeting of the Organization for Human Brain Mapping. Vancouver.

[hbm25274-bib-0036] Pluchino, S. , Muzio, L. , Imitola, J. , Deleidi, M. , Alfaro‐cervello, C. , Salani, G. , … Martino, G. , (2008). Persistent inflammation alters the function of the endogenous brain stem cell compartment Brain, 131, 2564–2578. 10.1093/brain/awn198 18757884 PMC2570715

[hbm25274-bib-0037] Preibisch, C. , Deichmann, R. , Preibisch, C. , Deichmann, R. , Preibisch, C. , & Deichmann, R. (2009). Influence of RF spoiling on the stability and accuracy of T1 mapping based on spoiled FLASH with varying flip angles. Magnetic Resonance in Medicine, 61, 25–135. 10.1002/mrm.21776 19097220

[hbm25274-bib-0038] Rocca, M. A. , Barkhof, F. , De Luca, J. , Frisén, J. , Geurts, J. J. G. G. , Hulst, H. E. , … Filippi, M. (2018). The hippocampus in multiple sclerosis. Lancet Neurology, 17, 918–926. 10.1016/S1474-4422(18)30309-0 30264730

[hbm25274-bib-0039] Rocca, M. A. , Longoni, G. , Pagani, E. , Boffa, G. , Colombo, B. , Rodegher, M. , … Filippi, M. (2015). In vivo evidence of hippocampal dentate gyrus expansion in multiple sclerosis. Human Brain Mapping, 36, 4702–4713. 10.1002/hbm.22946 26287572 PMC6869027

[hbm25274-bib-0040] Ropele, S. , Kilsdonk, I. D. , Wattjes, M. P. , Langkammer, C. , De Graaf, W. L. , Frederiksen, J. L. , … Fazekas, F. (2014). Determinants of iron accumulation in deep grey matter of multiple sclerosis patients. Multiple Sclerosis Journal, 20, 1692–1698. 10.1177/1352458514531085 24787429

[hbm25274-bib-0041] Salvoni, G. , Mohammadi, S. , Corbin, N. , & Ashburner, J. (2019). Impact of smoothing weights on voxel‐based quantification (VBQ) analysis, Paper presented at 25rd annual meeting of the Organization for Human Brain Mapping. Rome.

[hbm25274-bib-0042] Schmierer, K. , Scaravilli, F. , Altmann, D. R. , Barker, G. J. , & Miller, D. H. (2004). Magnetization transfer ratio and myelin in postmortem multiple sclerosis brain. Annals of Neurology, 56, 407–415. 10.1002/ana.20202 15349868

[hbm25274-bib-0043] Stonnington, C. M. , Tan, G. , Klöppel, S. , Chu, C. , Draganski, B. , Jack, C. R. , … Frackowiak, R. S. J. (2008). Interpreting scan data acquired from multiple scanners: A study with Alzheimer's disease. NeuroImage, 39, 1180–1185. 10.1016/j.neuroimage.2007.09.066 18032068 PMC2225446

[hbm25274-bib-0044] Stüber, C. , Morawski, M. , Schäfer, A. , Labadie, C. , Wähnert, M. , Leuze, C. , … Turner, R. (2014). Myelin and iron concentration in the human brain: A quantitative study of MRI contrast. NeuroImage, 93, 95–106. 10.1016/j.neuroimage.2014.02.026 24607447

[hbm25274-bib-0045] Tabelow, K. , Balteau, E. , Ashburner, J. , Callaghan, M. F. , Draganski, B. , Helms, G. , … Mohammadi, S. (2019). hMRI – A toolbox for quantitative MRI in neuroscience and clinical research. NeuroImage, 194, 191–210. 10.1016/j.neuroimage.2019.01.029 30677501 PMC6547054

[hbm25274-bib-0046] Vrenken, H. , Geurts, J. J. , Knol, D. L. , van Dijk, L. N. , Dattola, V. , Jasperse, B. , … Pouwels, P. J. (2006). Whole‐brain T1 mapping in multiple sclerosis: Global changes of normal‐appearing gray and white matter. Radiology, 240, 811–820. 10.1148/radiol.2403050569 16868279

[hbm25274-bib-0047] Weiskopf, N. , Suckling, J. , Williams, G. , Correia M. , Inkster, B. , Tait, R. , … Lutti, A. , 2013 Quantitative multi‐parameter mapping of R1, PD*, MT, and R2* at 3T: A multi‐center validation. Frontiers in Neuroscience. 7, 1–11. 10.3389/fnins.2013.00095 23772204 PMC3677134

[hbm25274-bib-0048] Zhao, C. , Deng, W. , & Gage, F. H. (2008). Review mechanisms and functional implications of adult neurogenesis. Cell, 132, 645–660. 10.1016/j.cell.2008.01.033 18295581

