# Supplementary material for: Voxel‐Based quantitative MRI reveals spatial patterns of grey matter alteration in multiple sclerosis
Source: Hum Brain Mapp. 2020 Nov 6;42(4):1003–12. doi: 10.1002/hbm.25274 (PMC7856642; doi:10.1002/hbm.25274)
Supplement: Supplementary file 1 — Table S1 Regions with significantly reduced gray matter MT saturation in MS patients compared to healthy controls. Tables S2: Regions with significantly reduced gray matter R1 in MS patients compared to healthy controls. Tables S3: Regions with significantly reduced gray matter R2* in MS patients compared to healthy controls. Tables S4: Regions with significantly reduced gray matter volume (VBM) in MS patients compared to healthy controls. Tables S5: Clusters where regional MT, R1 and R2* decrease with lesion fraction. Table S6: Clusters where cognitive score decreases with regional reduction of R1. [file HBM-42-1003-s001.pdf]

Table S1: Regions with significantly reduced grey matter MT saturation in MS patients compared to healthy controls.

Cluster-level		Voxel-level		MNI coordinates of local maxima			Side	Anatomical regions
Number of voxels	p-FWER corr.	F-value (1, 66)	p-FWER corr.					
**1987**	<0.0001	84.42	<0.0001	45	-18	6	R	Heschl's gyrus
		53.33	<0.0001	37	-21	0	R	Insula
**463**	<0.0001	40.10	0.009	25	-31	-4	R	Hippocampus
**166**	0.014	41.48	0.006	69	-23	3	R	Superior temporal gyrus
**107**	0.071	38.03	0.016	47	-14	-6	R	Superior temporal gyrus
**297**	0.001	30.47	0.139	38	8	-17	R	Superior temporal gyrus
**130**	0.037	30.40	0.142	28	52	7	R	Superior frontal gyrus
**127**	0.040	29.15	0.201	29	-59	51	R	Angular gyrus
**157**	0.017	36.99	0.022	38	-15	39	R	Precentral gyrus
**220**	0.004	39.38	0.011	-37	-28	10	L	Heschl's gyrus
**220**	0.004	36.36	0.026	-34	-23	1	L	Heschl's gyrus
**857**	<0.0001	40.09	0.009	-31	-27	-7	L	Hippocampus
**135**	0.032	37.70	0.018	0	-26	36	L	Cingulum
**189**	0.008	36.14	0.028	-43	-14	42	L	Postcentral gyrus
**120**	0.049	28.86	0.217	-34	-6	46	L	Precentral gyrus
**844**	<0.0001	33.38	0.061	0	-1	-9	L	Caudate

Tables S2: Regions with significantly reduced grey matter R1 in MS patients compared to healthy controls.

Cluster-level		Voxel-level		MNI coordinates of local maxima			Side	Anatomical regions
Number of voxels	p-FWER corr.	F-value (1, 66)	p-FWER corr.					
**319**	0.001	55.88	<0.0001	-46	3	-25	L	Middle temporal gyrus
**143**	0.030	45.57	0.004	-44	-52	19	L	Middle temporal gyrus
**727**	<0.0001	39.09	0.011	-33	-19	17	L	Insula
		35.14	0.035	-40	-27	9	L	Heschl's gyrus
**295**	0.001	37.56	0.018	-46	-60	-15	L	Inferior temporal gyrus
**153**	0.023	34.57	0.041	-52	-8	-5	L	Superior temporal gyrus
**130**	0.042	32.26	0.080	-34	-23	1	L	Insula
**1330**	<0.0001	48.75	0.001	-30	-31	-4	L	Hippocampus
**266**	0.002	26.60	0.375	-22	-54	-3	L	Lingual gyrus
**351**	<0.0001	43.91	0.003	47	-44	10	R	Middle temporal gyrus
**721**	<0.0001	35.18	0.035	27	-24	-9	R	Hippocampus
**132**	0.040	29.98	0.153	38	-30	45	R	Postcentral gyrus
**116**	0.062	35.29	0.034	38	-14	39	R	Precentral gyrus
**142**	0.030	27.45	0.303	0	-23	27	R	Cingulum

Tables S3: Regions with significantly reduced grey matter R2* in MS patients compared to healthy controls.

Cluster-level		Voxel-level		MNI coordinates of local maxima			Side	Anatomical regions
Number of voxels	p-FWER corr.	F-value (1, 66)	p-FWER corr.					
**331**	<0.0001	33.76	0.055	-55	-24	-32	L	Inferior temporal gyrus
**181**	0.009	31.95	0.092	-49	6	-28	L	Middle temporal gyrus
**136**	0.031	30.04	0.157	-42	-15	40	L	Postcentral gyrus

Tables S4: Regions with significantly reduced grey matter volume (VBM) in MS patients compared to healthy controls.

Cluster-level		Voxel-level		MNI coordinates of local maxima			Side	Anatomical regions
Number of voxels	p-FWER corr.	F-value (1, 66)	p-FWER corr.					
**1486**	<0.0001	129.82	<0.0001	38	-21	63	R	Precentral gyrus
**1509**	<0.0001	109.51	<0.0001	5	-12	-12	R	Thalamus
		78.59	<0.0001	18	-26	-9	R	Hippocampus
		64.73	<0.0001	5	-33	-9	L	Superior and inferior colliculi
**460**	<0.0001	71.79	<0.0001	41	-23	9	R	Heschl's gyrus
**421**	<0.0001	43.59	<0.0001	8	-81	-3	R	Lingual gyrus
**72**	0.027	41.27	0.03	15	-51	-2	R	Lingual gyrus
**333**	<0.0001	43.59	0.002	6	-84	17	R	Cuneus
**263**	<0.0001	36.17	0.013	33	9	-3	R	Putamen
**263**	<0.0001	36.17	0.013	33	9	-3	R	Putamen
**1467**	<0.0001	148.63	<0.0001	-27	-27	57	L	Precentral gyrus
**555**	<0.0001	57.83	<0.0001	-42	-21	5	L	Superior temporal gyrus
		48.23	<0.0001	-38	-24	11	L	Heschl's gyrus
**294**	<0.0001	42.81	0.002	-14	-95	-6	L	Lingual gyrus
**118**	0.004	27.90	0.146	-6	-89	2	L	Cuneus
**535**	<0.0001	39.93	0.005	-30	8	-9	L	Putamen
**65**	<0.0001	39.93	0.005	-30	8	-6	L	Putamen
**62**	0.043	27.87	0.148	-30	-59	50	L	Inferior parietal gyrus

Tables S5: Clusters where regional MT, R1 and R2* decrease with lesion fraction.

qMRI	Anatomical regions	Side	MNI coordinates of local maxima			Number of voxels	Local maximum F-value (1,66)	p-FWER corr.
MT	Medial superior frontal cortex	L	2	51	20	**172**	6.75	0.006
MT	Thalamus	R	14	-15	18	**1316**	6.08	<0.0001
	Caudate	R	13	9	20		5.85	
MT	Thalamus	R	2	-13	6	**416**	5.45	<0.0001
	Thalamus	L	1	-4	-7		5.00	
MT	Caudate	L	-7	7	8	**138**	5.35	0.018
MT	Thalamus	L	-10	-13	17	**116**	5.00	0.036
R1	Thalamus	L	-6	-29	4	**3643**	8.05	<0.0001
	Caudate	R	19	-6	24		7.40	
	Thalamus	R	2	-19	5		7.14	
R2	Thalamus	R	13	-14	18	**196**	6.12	0.006
R2	Anterior cingulum	L	-2	26	24	**160**	6.04	0.015

Table S6: Clusters where cognitive score decreases with regional reduction of R1.

qMRI	Anatomical regions	Side	MNI coordinates of local maxima			Number of voxels	Local maximum F-value (1,66)	p-FWER corr.
R1	Middle frontal gyrus, orbital part	R	39	56	-3	**126**	7.54	0.039
